# Parasites in the Pork Supply of Montreal

**Published:** 1883-02

**Authors:** 


### Popular Science Department.

PARASITES IN THE PORK SUPPLY OF MONTREAL.

The following are excerpts from an article, entitled as above, by Dr. Osler and Mr. Clement, in the January number of the *Canada Medical and Surgical Journal:*

So far as it is legitimate to draw deductions from the somewhat limited number of observations, we may say that trichinosis is a tolerably common affection in Canadian swine, though not nearly so frequent as in the neighboring States, still, it is much more so than is desirable in the interest of public health. Should microscopic examination of the flesh be included in the inspection, is a question which at once arises. In answering this, several circumstances must be taken into consideration. In the first place, although, per 1,000, a larger number of swine are infested here than in Germany, trichinosis in man is with us a very rare disease, while in Germany epidemics are of yearly occurrence. If we estimate that 100,000 hogs are killed annually for the local markets, that would give at least three or four hundred trichinous animals, whose flesh is consumed by the pork-eating members of the community. Then, about three and a half million pounds of American pork, representing about 15,000 hogs, have been imported into this city during the past year, and as in them the percentage of trichinæ is considerably higher than in Canadian animals, the probable number of infested carcasses consumed does not, at the lowest estimate, fall short of five hundred. Now, were the habits of the people of this city similar to those of the Germans, there can be no doubt that trichinosis, instead of being a rare affection, would be extremely common. Fortunately, raw or only partially cooked pork is not often eaten here, nor are the various kinds of sausages, so dear to the Teuton, much in vogue. *Knackwuerste* and *Bratwuerste*, forms of sausages which are very common, and which are eaten either raw or only warmed, have been the source of a large proportion of the known cases of trichinosis in Germany, 970 out of 1,267. People here almost invariably fry sausages, and smoked meats are not common, nor are they eaten without preliminary cooking. In short, the prophylaxis of the pot and oven in this country and in the neighbnring States does more for the public than the most stringent inspection, even as carried out in Prussia, where a microscopic examination is conpulsory. If thoroughly cooked, the trichinæ are killed, and may be eaten with impunity; and fortunately, there is a very widespread idea in the community that pork, in all forms, should be well cooked, and to this good custom may be attributed the immunity from infection which the public has enjoyed. Still, it is by no means pleasant to think of the quantity of trichinous flesh which is placed on our markets, and which probably exceeds the entire amount of pork confiscated for other causes. The difficulties in the way of systematic inspection are now, under the Abattoir By-Law, greatly lessened, but to subject the flesh of every hog killed to microscopic examination would require a staff of trained inspectors and an increased expenditure such as our civic authorities would not likely incur. Moreover, considering the rarity of cases of infection, it may be just as well to leave the matter to the cooks of the community, who have so long and so faithfully protected us, with this injunction, "See that all pork is thoroughly roasted, fried or boiled."

CYSTICERCUS CELLULOSÆ.

Of 1,037 hogs examined, 76 were infested—*i.e.*, 1 in 13.6. Only the livers were inspected, as it was impossible to examine the flesh thoroughly. The numbers varied from one or two to many dozens, and in most instances they were fully developed. The liver is more likely to be affected than the other parts, but the occurrence in this organ is a proof that the animal has been exposed, and should lead to a thorough examination of the flesh.

In order to obtain evidence of the extent to which "measled" meat produces disease—*i.e.*, tapeworm—in the community, we issued a circular to the city physicians asking the number of cases under treatment. Replies were returned by thirty-four doctors, who reported sixty-two cases. At the Smith Worm Company's office, Bleury street, about two new cases a week are treated; some of these, doubtless, come from the country, but we shall probably be within the mark if we estimate the number in the city as not far short of 200. How many of these are due to eating measley veal or beef, and how many to measley pork, we cannot say, but from the specimens examined it would seem that the beef tapeworm (*T. saginata*) is the more prevalent. Not that the pork measle is uncommon; the record above given shows just the contrary. To explain the greater frequency of *T. saginata*, we must suppose either that the beef measle occurs in greater proportion, or else the pork is more thoroughly cooked than the beef or veal. Then, too, much less pork is eaten fresh, and the salting and pickling processes are usually sufficient to destroy the measles. A point of interest is the temperature necessary to kill them. The observations of Professor Perronicito prove that they are invariably killed by a heat of 50 degrees C. or 122 degrees F. Indeed they were swallowed with impunity by his students after exposure to a temperature of 113 degrees F.

CONCLUSIONS.

1. The investigation shows that the hogs slaughtered for our markets present parasites in number sufficient to necessitate a more thorough inspection than is at present carried out.

2. As regards *Trichina spiralis*, which was found in the proportion of 1 to 250, we are of opinion that, considering the extreme rarity of cases of trichinosis, and the difficulties attendant upon a systematic inspection, a compulsory microscopic examination of the flesh of every hog killed is not at present called for.

3. In the case of "measles," the liver should be carefully examined, and if present in it, the flesh of the animal should receive the special attention of the inspector; if only in the liver, the entire carcass need not be confiscated.

4. Echinococcus cysts in the liver render that organ unfit for food, but in other parts, unless very numerous and disorganizing, they may be cut out, and the carcass remain marketable.

5. The public should be made aware of the possible dangers of eating, in any form, raw or partially cooked meat. The best safeguard against parasitic affections is not so much inspection of the flesh, unless, indeed, this is minutely carried out, as careful attention to culinary details.

6. To reduce the number of infested hogs, greater attention should be paid to their hygienic surroundings, particularly in the matter of feeding. The danger is not during the period when the animals are penned and fed on grain, &c., but when they are allowed to roam at large and feed indiscriminately.

---

Women physicians have been refused permission to practice in Austria.

